# Fine-tuning the extent and dynamics of binding cleft opening as a potential general regulatory mechanism in parvulin-type peptidyl prolyl isomerases

**DOI:** 10.1038/srep44504

**Published:** 2017-03-16

**Authors:** András Czajlik, Bertalan Kovács, Perttu Permi, Zoltán Gáspári

**Affiliations:** 1 Pázmány Péter Catholic University, Faculty of Information Technology and Bionics, Budapest, Hungary; 2 University of Jyvaskyla, Dept. of Biological and Environmental Science, Dept. of Chemistry, Nanoscience Center, Jyvaskyla, Finland

## Abstract

Parvulins or rotamases form a distinct group within peptidyl prolyl cis-trans isomerases. Their exact mode of action as well as the role of conserved residues in the family are still not unambiguously resolved. Using backbone S^2^ order parameters and NOEs as restraints, we have generated dynamic structural ensembles of three distinct parvulins, SaPrsA, TbPin1 and CsPinA. The resulting ensembles are in good agreement with the experimental data but reveal important differences between the three enzymes. The largest difference can be attributed to the extent of the opening of the substrate binding cleft, along which motional mode the three molecules occupy distinct regions. Comparison with a wide range of other available parvulin structures highlights structural divergence along the bottom of the binding cleft acting as a hinge during the opening-closing motion. In the prototype WW-domain containing parvulin, Pin1, this region is also important in forming contacts with the WW domain known to modulate enzymatic activity of the catalytic domain. We hypothesize that modulation of the extent and dynamics of the identified ‘breathing motion’ might be one of the factors responsible for functional differences in the distinct parvulin subfamilies.

Peptidyl prolyl isomerases (PPIases) catalyze the isomerization of the peptide bond preceding proline residues. PPIases play an important role not only in protein folding but also in the regulation of several of biological processes like chromatin remodeling, transcription and nuclear receptor signaling^1^. They can be divided into three non-homologous and structurally different families, known as cyclophilins, FKBPs (FK506 binding protein and its relatives), and parvulins. The latter, highly conserved subfamily consists of small (~10 kDa) proteins that are present in both pro- and eukaryotes^2^. Their structure consists of a four-stranded antiparallel β-sheet surrounded by four α-helices (αβ3βαβ2, parvulin fold)^3^. Parvulins play key roles in many important biological processes including the cell-cycle regulation, apoptosis and protein quality control^4^,^5^. Due to these functions, they are involved in neurodegenerative disorders such as Parkinson’s and Alzheimer’s disease^6^,^7^ as well as various cancers^8^,^9^,^10^,^11^.

The two main classes of parvulins are the Pin1-type and non-Pin1-type parvulins. For the first group the isomerization reaction is phosphorylation-dependent, that is, Pin1-type parvulins selectively recognize either Ser-Pro or Thr-Pro sequences where the serine/threonine is phosphorylated. Most of them, like their archetype Pin1, contain an N-terminal WW domain responsible for a ligand recognition and a conserved C-terminal PPIase domain with a phosphate-binding loop. Interestingly, there are some known members of the Pin1 family that do not possess a WW domain, i.e. some plant Pin1 proteins^12^ and TbPin1 from *Trypanosoma brucei*^13^. In contrast, the non-Pin1-type parvulins are single domain proteins and their isomerization mechanism is phosphorylation-independent. Thus, the phosphate-binding site is missing, which is the only significant structural and functional difference in the PPIase domain between the Pin1-type and non-Pin1-type parvulins.

The exact mechanism of action of PPIases is not yet elucidated. It is unclear whether different PPIase families or distinct members within a family exhibit similar mechanisms. However, it is generally accepted that there is no breaking and reforming of the peptide bond, thus, the bond is converted from the *cis* to the *trans* form via rotation through a twisted amide intermediate^14^. In a recent study on cyclophilin A, dynamic structural ensembles were generated using chemical shift data for a structurally heterogeneous state where both the *cis* and *trans* isomer of the ligand are present. Analysis of the electrostatic field within the ligand binding site suggested an ‘electrostatic handle’ mechanism, speculated to be also valid for other PPIases^15^. Furthermore, although the active site of the parvulin-type PPIases is well-defined, the mechanistic role of the constituent residues is not yet fully clarified.

The two highly conserved histidines of parvulin-type PPIases have been suggested to be important for catalysis. However, many His mutants of Pin1 retained PPIase activity and, interestingly, the selectivity of Pin1 towards phosphorylated substrates was dependent on the identity of the replacing residues. Circular dichroism investigations together with proteolytic susceptibility data led to the suggestion that the mutations influenced the dynamics of Pin1 rather than causing substantial structural rearrangements^16^.

The high-resolution structure of human Par14 offered insights into a hydrogen-bonding network involving the two histidines as well as a threonine and an aspartate at the ‘outer edges’ of the imidazole side chains. Replacing the threonine with alanine in Pin1 resulted in 300-fold decrease in catalytic activity while not compromising structural integrity^17^. The aspartate is in a position occupied by a cysteine in Pin1, also suggested to be important in catalysis earlier^18^. Detailed theoretical studies hinted that this cysteine, through changes in its protonation state, can mediate dynamic changes in this network^19^. Indeed, replacing this cysteine with alanine or serine caused the disruption of the hydrogen bond between the histidines^20^.

NMR analysis of conformational exchange in Pin1 suggested a link between motional modes present in the catalytic domain and the rate of catalysis, leading to the hypothesis that the internal motions assisting catalysis are an intrinsic feature of Pin1^21^. Ligand binding has been shown to influence the internal dynamics of Pin1, leading to more extensive contact between the PPIase and WW domains proposed to be linked to the loss of flexibility at specific conserved hydrophobic sites^22^. Specifically, changes in side-chain mobility upon ligand binding highlighted the role of an internal conduit consisting of hydrophobic side-chains. These residues are conserved in Pin1 homologs and have been suggested to play an important role in inter-domain communication^23^. More recent studies showed that the role of the WW domain negatively regulates PPIase activity in Pin1^20^.

Molecular dynamics studies of Pin1 revealed allosteric pathways and suggested that substrate binding by the WW domain leads to preorganization of the catalytic site^24^. The range of identified residues participating in allosteric communication extends those revealed by NMR studies of side-chain flexibility^23^. Importantly, the preorganization was identified as a closure of the loop regions surrounding the substrate-binding cleft, and the presence of the WW domain enhances the flexibility of these loops^24^. A recent study combining NMR spectroscopy and molecular dynamics indicated that the WW domain undergoes structural changes upon ligand binding and these changes affect its association with the PPIase domain in full-length Pin1, a mechanism proposed to be responsible for different activity of Pin1 on ligands with single and multiple recognition sites^25^.

To get further insights to the differences between various parvulins, we have combined molecular dynamics simulations with experimentally available backbone S^2^ order parameters to conduct a comparative analysis of three single-domain parvulins. One of these (TbPin1) is Pin1-type parvulin lacking a WW domain and the other two (SaPrsA, CsPinA) are non-Pin1-type parvulins.

SaPrsA from *Staphylococcus aureus* is responsible for folding of secreted proteins. Although member of a different subclass, its three-dimensional structure and active site arrangement proved to be almost the same as for human Pin1. The profound knowledge of histidine protonation states of His residues was investigated in detail experimentally obtained, revealing different tautomeric states for the two conserved histidines and the presence of a hydrogen bond between their side chains^26^ and this is also reflected in the corresponding PDB structure (id: 2JZV).

TbPin1 from *Trypanosoma brucei* is considered as a putative Pin1-type parvulin despite it lacks the WW domain^13^. It was shown that replacing Cys65 (corresponding to Cys113 in Pin1) with Ala diminishes its catalytic activity, in accordance with other studies on the role of this residue (see above). In the structures deposited in PDB (id: 2LJ4) both His residues are protonated and there are no hydrogen bonds between them.

CsPinA from the psychrophilic archaeon *Cenarchaeum symbiosum* has been shown to possess an atypically large peptide-binding site. Similarly to TbPin1, the two histidines are protonated in the available PDB structure^27^.

The three investigated parvulins share a common structural core (Fig. 1) where the ligand-binding cleft is flanked by two lobes. The smaller lobe, shown in the left of the figure and closer to the N-terminus, consists of a short helix and a loop structure, whereas the larger one is formed by a four-stranded antiparallel β-sheet and two helices located opposite the cleft. The conserved histidines are located in the two central strands of the β-sheet. Notably, all residues forming the hydrogen-bonding network described above can be found in the large lobe (Fig. 1). Available backbone S^2^ order parameters for the three molecules suggest some characteristic differences with TbPin1 showing the lowest average values (Fig. 2).

## Results and Discussion

### Restrained ensembles resemble the native structures and are restricted relative to unrestrained ones

For all three parvulins, SaPrsA, TbPin1 and CsPinA, restrained ensembles were generated according to the MUMO protocol^28^ using backbone S^2^ order parameters and NOE distance restraints. As a control, unrestrained ensembles were also generated (see Methods for details). The MUMO and unrestrained ensembles contain 728 conformers each.

In the case of the MUMO ensembles, correspondence to S^2^ order parameters could be achieved without compromising the agreement with Cα and Hα chemical shifts that are most sensitive to protein structure (Table 1). The unrestrained ensembles, similarly to those deposited in the PDB, are not compatible with the backbone S^2^ data, as can be expected^29^.

For CsPinA, the S^2^ value of the C-terminal residue, Gly97, had to be excluded from the backbone S^2^ correlation because of a conformational drift during the MD simulation resulting in two alternative orientations of this residue in the final ensemble. Closer analysis hints that this might be the consequence of the NOE restraints in the region (exclusion of all restraints violated in the PDB ensemble hinders the occurrence of the conformational drift). However, as this region is not included in any of the consensus mappings, this does not affect any of our conclusions below.

It should be noted that in our calculations NOE data were used to restrain the ensemble close to the native conformation, but, as in other ensembles reflecting multiple NMR-derived parameters, it can not be expected that all NOE restraints are fulfilled^30^,^31^. The ratio of violated restraints is below 1% for all three molecules in this treatment (Supplementary Table S3).

Both the MUMO and the unrestrained ensembles are clearly more diverse than the PDB-deposited ones, with the restrained ones being conformationally more restricted, as evidenced by PCA analysis (Fig. 3) and RMSD values in Table 1. In general, the PDB-deposited ensembles sample only a subset of the conformational space occupied by the MUMO ensembles and, with the notable exception of CsPinA, the MUMO ensembles correspond to a subset of the unrestrained ones (Fig. 3). This trend is more evident when only structurally equivalent residues, defined in the basis of a structural alignment of the three proteins (see Methods), are considered.

### The three parvulin ensembles differ in the extent of binding cleft opening

**Figure 1:**
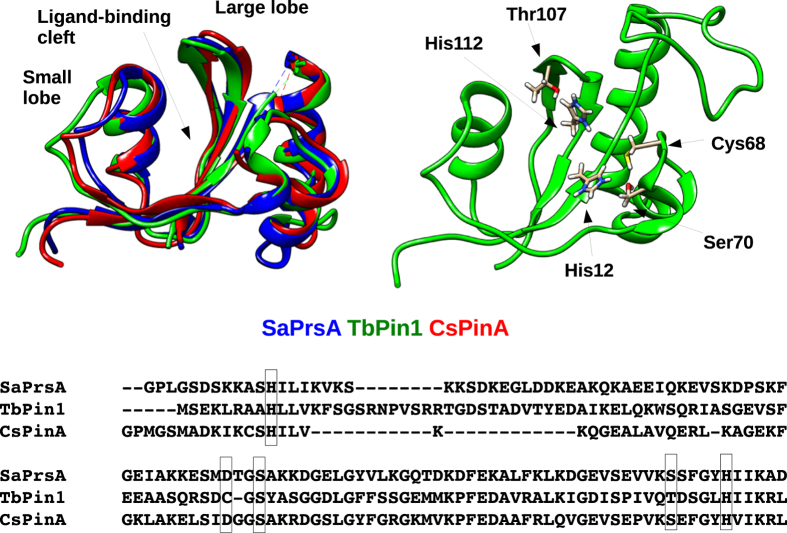
Top left: superimposed Cα trace of SaPrsA (blue), TbPin1 (green) and CsPinA (red) showing only the residues aligned by MAMMOTH-Mult. Top right: TbPin1 structure with the residues involved in the hydrogen-bonding network highlighted. Bottom: sequence alignment of the three parvulins with the residues involved in the hydrogen-bonding network highlighted.

**Figure 3:**
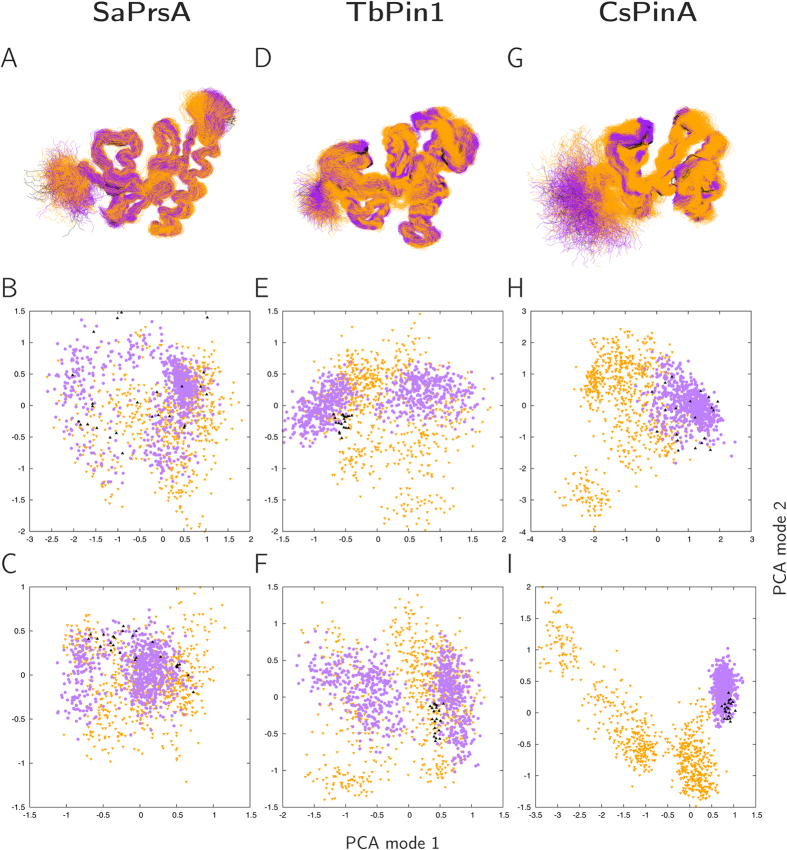
Top panels (**A,D,G**): structures superimposed with MOLMOL (black: ensembles deposited in the PDB, orange: unrestrained ensembles, purple: MUMO ensembles). Middle panels (**B,E,H**): PCA plots (first two modes) of the full structures. Bottom panels (**C,F,I**): PCA plots (first two modes) of the residues corresponding to the consensus mapping of the three proteins (see Methods for details). Black triangles: deposited PDB ensembles, orange triangles: unrestrained ensembles, purple circles: MUMO ensembles.

**Figure 4:**
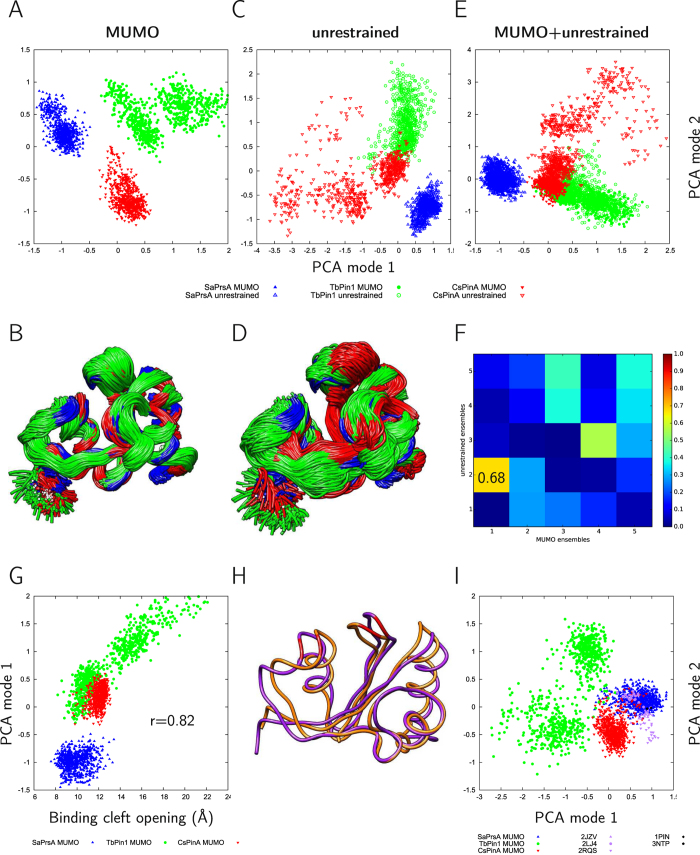
Diversity of the ensembles using only the positions common to all three parvulins. (**A**) PCA analysis (modes 1–2) and (**B**) structure superposition of the combined MUMO ensembles, (**C**) PCA analysis and (**D**) superposition of the combined unrestrained ensembles, (**E**) PCA analysis of the combined MUMO + unrestrained ensembles, (**F**) overlap of the first 5 PCA modes of the MUMO and unrestrained ensembles shown in (**A** and **C**); (**G**) correlation of the first PCA mode in the MUMO ensembles with the binding cleft opening defined by the distance between the Cα atoms of residues 86 and 109 (2LJ4 numbering); (**H**) average conformer in the MUMO ensemble distorted along the PCA mode 1 to show the opening motion, residues 86 and 109 (2LJ4 numbering) highlighted; I) PCA analysis of selected atoms in the binding site of the molecules (see Methods), PCA mode 1 here is also dominated by the opening-closing motion of the binding cleft.

The ensembles of the three different PPIase domains were compared using the set of residues that could be aligned in a multiple structural alignment (see Methods). The resulting mapping contains 89 residues including the substrate binding cleft and the two surrounding lobes (Fig. 1). PCA analysis of the combined ensembles reveals that they occupy distinct regions of the conformational space according to the first two modes covering 44 and 25% of the variability of the structures, respectively (Fig. 4A,B). The same remains mostly valid for the unrestrained ensembles (Fig. 4C,D, with the first two modes covering 36 and 24% of the variability) and even when the MUMO and unrestrained ensembles are analyzed together (Fig. 4E).

PCA mode 1 in the MUMO ensembles largely overlaps with PCA mode 2 of the unrestrained ones (Fig. 4F). Closer analysis of this mode reveals that mode 1 in the MUMO ensembles reflect a motion roughly corresponding to the opening and closing of the substrate binding cleft and can be approximated by measuring the distance between residues near the tip of the two flanking loops of the cleft (Fig. 4G,H). Along this coordinate, the TbPin1 ensemble occupies the largest region, thus, our analysis suggests that this motion is primarily present in the TbPin1 ensemble but is also clearly present in SaPrsA. Nevertheless, the deviations between the ensembles can be primarily attributed to the differences in binding cleft opening in parvulins, termed ‘breathing motion’ hereafter. We have analyzed the residues involved in ligand binding and largely conserved in all three parvulins analyzed (see Methods). PCA analysis of selected heavy atoms is shown in Fig. 4I. PCA mode 1 largely describes the alterations of the distance of residues located at opposite sides of the binding cleft, most prominently those corresponding to Met130 and Cys113 in Pin1. Thus, the differences observed in the binding sites can also mostly be attributed to the opening-closing motion separating the full structures in the MUMO ensembles.

S^2^ restraining yields a conformational ensemble consistent with the fast (ps-ns) internal motions, thus, it is expected that the resulting ensemble samples the conformational space around an average structure representing the native state. However, in the case of TbPin1, the nature of the conformational movements sampled, in particular the breathing motion, would be expected to occur on a slower time scale. Thus, we regard the generated ensembles as reflecting the upper limit of the conformational space sampled by the three parvulins during their fast motions. In this interpretation the ensembles do not necessarily reflect that the binding site opening - at least to the extent reflected by the TbPin1 ensemble - indeed occurs on such a fast time scale, although the correspondence to S^2^ order parameters strengthens the validity of larger motions in TbPin1 along this mode than in the other two parvulins.

In principle, S^2^ restraining does not necessarily restrict the extent of the motions sampled but limits primarily only their directions. Interestingly, RMSD values suggest that the TbPin1 ensemble is not more diverse than the SaPrsA or the CsPinA ensemble in general. Considering the results of PCA analysis it can be safely stated that its diversity is distributed along different internal motions than observed for the other two molecules. It should also be noted that for Pin1, conformational motions expected to be characteristic of slower time scales also occurred in a 100 ns simulation^24^. As both Pin1 and TbPin1 act on phosphorylated substrates, this observation - relatively large amplitude motions of the binding cleft at a fast time scale - might even have relevance for this subtype of parvulins. For TbPin1, NMR relaxation analysis revealed a group of residues with slow exchange located at the phosphate-binding loop, which might also indicate the presence of larger-scale opening-closing motions, although on a slower time scale.

### Comparison with other parvulins highlights diversity in the hinge region

To compare the ensembles with other parvulin domains of known structure, we have generated a consensus residue mapping between 100 rotamase domains, including the representative structures of SaPrSA, TbPin1 and CsPinA, available in the PDB (Supplementary Fig. S6). Interestingly, this consensus mapping contains only 53 residues including only one of the conserved histidine residues, as the one closer to the N-terminus is not part of this consensus. We have performed PCA analysis on the three MUMO ensembles plus the 100 rotamase domains.

It is somewhat surprising that, contrary to expectations^32^,^33^, the diversity of the MUMO ensembles is higher than that of the different PDB-derived parvulins. However, at least for structures determined with crystallography it is expected that the crowded environment of a crystal does not favor open conformations.

**Figure 5:**
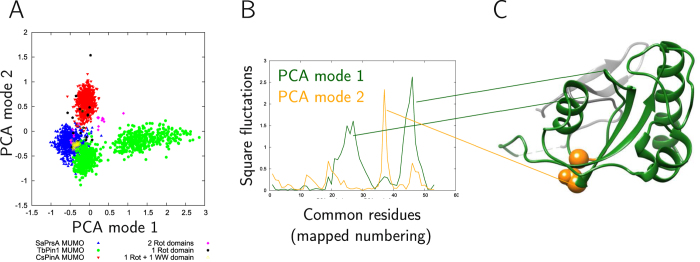
(**A**) PCA analysis of all PPIase domains in the PDB using the positions common to all such domains. (**B**) Displacements along PCA mode 1(dark green) and 2 (orange). Note that the residue numbering refers to the common positions comprising 53 residues only. (**C**) Location of the residues with largest displacements in PCA mode 2 projected to the full PIN1 (in orange, Gly144 and Glu145). Note that the parts retained based on the structure alignment contain only the two loops connected with the two peaks in PCA mode 1. The WW is domain is in the back, colored light gray.

Similar to the results obtained from comparing the MUMO ensembles only, PCA mode 1, covering 48% of the variability of the structures, corresponds to the opening and closing of the substrate binding site. Interestingly, the only group with a substantial distribution along this mode corresponds to the proteins with 2 rotamase domains (Fig. 5A). We note that from these, only one available structure, 1m5y (*E. coli* SurA, an outer membrane protein chaperone) contains both parvulin domains, and the two domains in all four chains of this PDB entry are well separated along the 1st PCA coordinate, with N-terminal domains having a positive first coordinate and C-terminal domains exhibiting a negative one in the plot (Fig. 5A). In this structure, the first (N-terminally located) parvulin domains are surrounded by an extension around the large lobe of the binding cleft.

Strikingly, in this analysis the MUMO ensembles of the three parvulins investigated are separated along PCA coordinate 2, which describes differences near the ‘bottom’ of the peptide binding cleft, regions acting as linkers between the two lobes around the binding cleft. The hinge region identified in PCA mode 1, defined by the minimum around residue 38 in the mapped numbering (Gly144 in Pin1), is mostly affected by displacements along PCA coordinate 2 (Fig. 5B). A similar motion is responsible for the separation of the MUMO ensembles along PCA mode 2 when analyzed without additional structures (Fig. 4A).

As the different parvulin ensembles are also separated along this motional mode, it is tempting to assume that they correspond to different states along a common motional mode occurring on a slower time scale and some of their functional differences can be explained by the differences required in their ligands to trigger proper binding and effective catalysis to occur. However, whether larger-scale motions of this type occur in all molecules and whether they can be linked to any aspect of catalysis remains to be shown. Our first-approximation estimate of the electrostatic field at the position of the carbonyl C of the isomerised amide bond of the substrate did not reveal any dependence on the extent of the opening of the binding cleft (not shown). However, the difference between the mode and extent of this opening motion between these molecules might still be valid and might influence their functional diversity.

In Pin1, the WW domain exerts a significant influence on the catalytic properties: Both WW-deletion mutants and point mutants influencing the interaction between the WW and parvulin domains show markedly different activity relative to wild-type Pin1. As the WW-parvulin interaction site is located on the side of the ligand-binding cleft, it can be speculated that the extent and mode of interaction with the WW domain modulates the breathing motion of the cleft, thereby contributing to the regulation of its enzymatic activity. This hypothesis is consistent with the recent molecular dynamics study of Pin1, where the presence of the WW domain enhanced the flexibility of the loops around the binding site^24^ and also with the separation of the WW-containing and WW-less parvulins in our comparative PCA analysis. These parvulins are separated along PCA mode 2 corresponding to differences distributed near the bottom of the binding cleft, ideal in a position to modulate the flexibility of the parvulin domain. This is supported by our observation that the hinge region identified for the opening-closing motion displays the highest displacement in the PCA analysis of all PPIase domains with known structure (Fig. 5C). Thus, we hypothesize that changes in the hinge region, either caused by mutations or the interaction with the WW domain, if present, can influence the opening of the substrate binding cleft. Indeed, residues adjacent to this putative hinge region have been shown to be influenced by mutations introduced into the WW domain of Pin1^20^. Moreover, NMR relaxation analysis identified two residues in this region, Ile 98 and Asp100 (according to residue numbering in 2LJ4) that are involved in slow conformational exchange^13^.

### Possible role of the conserved histidines and the hydrogen bond network

**Figure 6:**
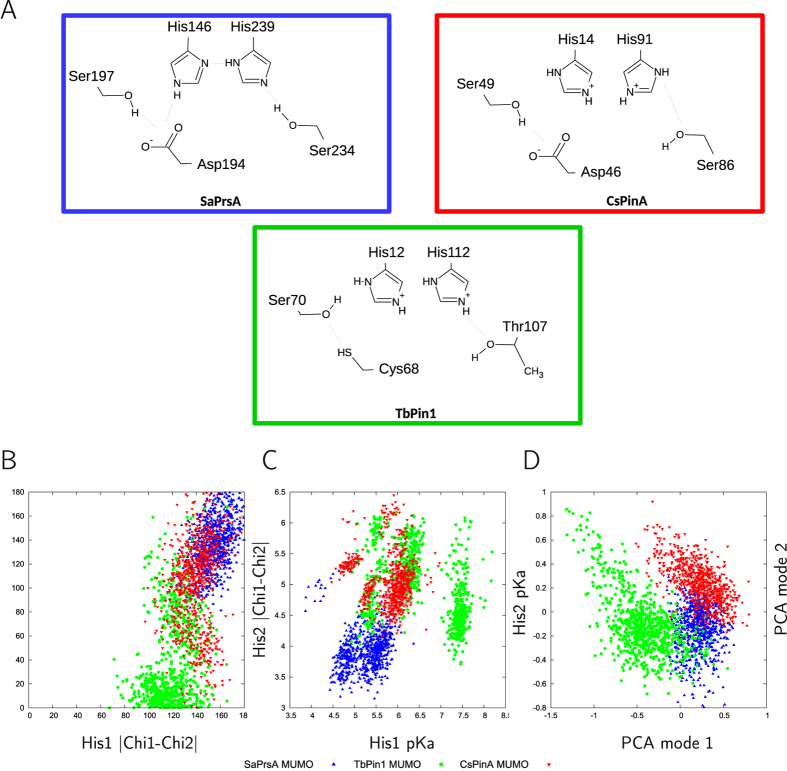
(**A**) Scheme of the residues involved in the proposed hydrogen bond network. The orientation of the histidines was taken from the first model of the PDB-deposited ensembles. Residue numbering corresponds to that of the PDB ensembles. Hydrogen bonds detected in the MUMO ensembles are shown. Note that the occurrence of the individual hydrogen bonds varies in the ensembles. (**B**) Chi1-Chi1 and Chi2-Chi2 differences between the two conserved histidines indicate different relative orientations of the side chains in the three ensembles. (**C**) PropKA-predicted pKa values of the histidines in the SaPrsA (blue), TbPin1 (green) and SaPrsA (red) ensembles. (**D**) PCA analysis of the CA positions of the 5 residues involved in the hydrogen bond network in the three MUMO ensembles. Mode 1 is dominated by relative displacements of the flanking Ser/Thr and Cys/Asp residues, whereas mode 2 describes the reorientation of the two central histidines. Overall, PCA coordinate 1 here shows a correlation of 0.61 with first PCA mode in the full MUMO ensembles corresponding to the opening-closing motion of the binding cleft.

In search for the factors that might influence the different preferences of the three parvulins with respect to the opening of the binding cleft, we performed detailed analysis of the conformation, possible pKa value distribution and hydrogen-bonding pattern of the conserved histidine residues. Both the conformation, as measured by the side-chain chi1 and chi2 torsions and the distribution of the predicted pKa values are different in all three ensembles. It is important to note here that both TbPin1 and CsPinA were calculated with fully protonated histidine side chains, consistent with the PDB-deposited structures, whereas for SaPrsA the results of the detailed analysis published along with the structure^26^ were taken into account. Thus, in SaPrsA there is a hydrogen bond between the two His residues that can not form in the two other parvulins due to the protonation state and conformation of the imidazole rings (Fig. 6A). This is also reflected in the observation that the relative side-chain orientations of the histidine side chains are most restricted relative to each other in the SaPrsA ensemble (Fig. 6B).

It should be noted that although the pKa values of histidine residues are conformation-dependent^34^, the pKa values predicted here probably overestimate the actual variability occurring during fast motions (Fig. 6C).

Analysis of the CA positions of the residues involved in the putative hydrogen-bonding network suggest that there are motions confined within the large lobe that are at least weakly correlated with the breathing motion of the full parvulin molecules, again dominated by the motions TbPin1 (Fig. 6D). Although the two data sets are not independent (i.e. the residue-specific analysis uses a subset of CA atoms used for the global one) and correlation does by no means indicate causation on its own, our results are compatible with a scenario where the dynamic hydrogen bond proposed by Barman and Hamelberg^19^ modulates the mobility of the β-strands of the large lobe relative to each other, precluding the small rearrangements coupled to the opening motion. Thus, changes in His protonation and thus the formation of the hydrogen bond might be one of the factors to be modulated by exact spatial context including substrate binding and interaction with the WW domain^23^,^24^. Besides the formation of a hydrogen bond between the two imidazole moieties, His protonation also affects the conformational preferences of His residues that might provide even more subtle ways of regulation in this respect. The lack of the hydrogen bond in TbPin1 and CsPinA is consistent with the observation that Pin1 histidine mutants were catalytically active^16^ and supports the speculation that His protonation state can be a relevant factor in modulating activity. Testing of this hypothesis would require carefully designed *in vitro* experiments. Moreover, it was noted that the level of isomerase activity on phosphorylated substrates could be modulated by replacing the histidine residues. Among the parvulins investigated here, TbPin1 is a WW-domain-less parvulin exhibiting a Pin1-type PPIase acting on phosphorylated substrates. The above observations together with our findings are consistent with a model where the histidines play pivotal role is in modulating the dynamics of parvulin-type PPIases as suggested by Bailey and coworkers^16^ and that this contributes to substrate binding. Thus, even if the histidine protonation states are not physiologically relevant as modeled here, the general mechanism, i.e. that the protonation state can influence the extent and nature of the breathing motion, might still be valid. Such a role of histidine residues would not be unprecedented as a recent study suggested the protonation state of a His side chain can modulate loop flexibility and ligand release in Langerin, a C-type lectin receptor^35^.

### A general model for parvulin specialization and regulation based on the extent and dynamics of the opening of the substrate binding cleft

We propose a general model for parvulin-type PPIases where the extent of opening and the breathing motion of the substrate binding cleft plays a role in substrate selectivity and catalysis. Both of these factors, namely, the preferred geometry of the binding cleft and the dynamics of the breathing motion might be modulated by different factors, such as interactions with the substrate, a WW domain (if present) and the state of the hydrogen bond network connecting the strands of the large lobe. These factors might not be independent from each other, but the connections between the conduit formed by hydrophopic residues with the hydrogen bond network are yet to be established. Molecular dynamics simulations of Pin1 also suggested that the closure and flexibility of loops around the binding site are influenced by interactions with the WW domain and dependent on protein-substrate interactions at both the PPIase and the WW domain^24^,^25^.

Our model, however appealing, is a necessary simplification but provides a testable framework for future investigations. A recent paper has indicated the role of binding site dynamics in substrate binding and catalysis in an FKBP-like PPIase^34^. We note here that modulation of the ligand-binding site opening dynamics has been implicated in the evolution of GK domain proteins^37^, thus, similar mechanisms, if valid, might represent a general way to fine-tune protein function.

## Methods

### Generation of dynamic structural ensembles

Calculations were performed using an in-house modified version of GROMACS 4.5.5^38^ capable of handling S^2^ order parameter restraints^29^,^39^. The coordinates and NOE restraint lists for structures 2LJ4, 2JZV and 2RQS were obtained from the RCSB web site. S^2^ order parameters were obtained from the original authors of the structures for 2JZV and 2LJ4, and from BMRB (entry 11080) for 2RQS. NOE lists were converted to a format similar to the one used in the distance restraint section of GROMACS topology files. In order to ensure standardized treatment of NOE lists and to ensure compatibility with the restraining scheme in GROMACS (r^−6^ averaging), a filtering procedure was used to select the NOE distances used for restraining. First, all stereospecific assignments were rewritten as ambiguous by listing all possible atom pairs. This ensures that no erroneous stereospecific assignments remain. After removing redundant restraints that might be generated with this step, the distances were checked against the original, PDB-deposited ensembles and those violated over 0.5 Å were removed. Finally, the remaining distances were converted to binned ones, corresponding to three categories, 2.5–3.5, 3.5–5.0 and 5.0–6.0 Ångstroms. This list was used in the following calculations to ensure that the structures remain close to the native state during the restrained simulations.

Using the prepared NOE and S^2^ restraints^39^, calculations were performed in a way similar to the MUMO protocol^28^ with 8 replicas and NOE restraining over neighbouring ones. The AMBER99SB force field was used with the explicit water model TIP3P. Simulations were run for 10 ns (totaling to 8 × 10 = 80 ns simulation time for each molecule), and after discarding the first 1 ns, structures were saved every 100 ps, resulting in a total of 728 conformers for all three molecules. As a control, unrestrained simulations were run with exactly the same setup but with S^2^ and distance restraint force constants set to zero. Correspondence of the ensembles to S^2^ parameters was checked with the CoNSEnsX server^40^. Chemical shifts were back-calculated with shiftx2^41^ with parameter settings corresponding to the experimental conditions for each molecule as described in the original publications (using default settings resulted in only minor changes not affecting our conclusions). NOE distances were checked using an in-house script using the same calculation scheme as GROMACS.

### Comparative structural analysis of the ensembles

To be able to do direct comparisons between the two ensembles, we have generated a structural alignment of the annotated representative models (MODEL 1 in each case) of the 2JZV, 2LJ4 and 2RQS structures with MAMMOTH-Mult^42^. Using this alignment we have prepared a consensus mapping of the residues and used this to generate PDB files containing only the residues aligned by MAMMOTH-Mult and have used a consensus residue numbering based on this alignment. The structural ensembles were merged with the original PDB-deposited conformers using this mapping (denoted three-way consensus thereafter) and the resulting ensemble was subjected to PCA analysis using ProDy^43^,^44^.

For a wider comparative analysis involving other PPIase domains, we searched sequences in the PDB^45^ (pdb_seqres.txt, downloaded on 6 May 2015) with hmmscan using the rotamase HMM profile (Pfam ID PF00639.16). The full protein sequences corresponding to the chains with a PPIAse domain were obtained from UniProt and again scanned for the presence of PPIase and WW domains using hmmsearch^46^. Coordinates of the PPIase domains in the protein chains as identified by the HMM search were superimposed using MAMMOTH-Mult and this alignment was used to generate a consensus residue mapping. We refer to this mapping as the core region, common to all parvulin-type PPIases analyzed here. This mapping provided the basis for generating PDB files for all structures containing only the aligned residues with consensus numbering. The structures were then superimposed with MOLMOL^47^ and several outliers (all belonging to proteins with one PPIase and no WW domain) were removed. This structure set was then merged with PDB files corresponding to the calculated structural ensembles mapped according to this “wide consensus” and the resulting ensemble was subjected to PCA analysis using ProDy. Displacements along PCA modes were extracted from the nmd files generated by ProDy. Comparison of motional modes was performed as described by Meireles *et al*.^48^. The binding site openness was defined with the distance between the Ca atoms of residues Met86 and Ser109 in TbPin1, and the corresponding residues in the generated ensembles.

### Analysis of the ligand-binding site

Atoms participating in ligand binding and common to all three investigated structures were identified as follows. Two X-ray structures of Pin1:ligand complexes were used, 1PIN^18^ and 3NTP^14^. These two structures contain different ligand types, thus, a set of atoms that can be regarded as common between the two ligands had to be defined first. After visual inspection, 9 such atoms were chosen: from the part corresponding to the proline residue, the ring atoms including the alpha carbon, the amide N, carbonyl C, and from the part corresponding to the preceding residue (Ala in 1PIN), three C atoms corresponding to the C, CA and CB atoms.

Protein atoms closer than 7 Å to any of these identified 9 ligand atoms were identified and only those common in the two Pin1 structures were retained. In the next step, a MAMMOTH-MULT alignment of the 1PIN, 3NTP, 2JZV, 2LJ4 and 2RQS structures (using the first models from the original NMR ensembles) was generated and based on this mapping, atoms common in all 5 structures were retained. After inspecting the common set of atoms and residues, where the residue type did not match between the structures but the atom type was the same, the atom was retained. Atoms not common between all 5 structures were omitted, the only exception being Cys 113 (1PIN numbering), a residue proposed to be important in catalysis and replaced by Asp in 2JZV and 2RQS, in which cases the Asp CG atom was considered instead of the Cys SG. This resulted in 38 atoms altogether from 10 residues including the two conserved histidines and all residues that were used as a common set of binding site atoms shared by the 5 parvulins. Although this approach contains subjective elements, we expect that the size of the set and the included residues warrant that the results obtained have valid implications.

### Calculation of the electrostatic field

With correspondence to the work of Camilloni *et al*., electrostatic field was computed in the binding site of the generated ensembles^15^. According to the catalytic mechanism proposed by Camilloni *et al*., a large Z-component of the electrostatic field in the position of the proline carbonyl carbon atom (given that the amide plane coincides with the x-y plane) facilitates cis-trans isomerisation of the proline peptide bond. In order to obtain the values of the electrostatic fields, all ensembles were rotated in a common frame. The common frame was given by pin-1 protein (PDB: 1PIN). Its ligand (Ala-Pro) was rotated in a way that the N, N-CD and N-CD-CA atoms of the proline coincide with the origin, the x axis and the x-y plane, respectively. Afterwards, all the replicas of the generated ensembles were superimposed to the properly rotated pin-1 structure. Prior to the superposition, multiple sequence and structure alignment was done with MAMMOTH-MULT for the original pin-1 structure and the generated structures of SaPrsA, TbPin1 and CsPinA, to identify the overlapping segments. For the superposition only Ca atoms were used that were close to the binding site (residues 115–163 in 1-pin, and corresponding residues in the other molecules). The electrostatic field was computed in the atomic positions of the ligand, based on the partial charges in the topology file generated by GROMACS. For calculating the electrostatic field, only atoms within the cutoff range and with partial charges higher than the charge cutoff were taken into account. To verify the robustness of the results, the electrostatic field was determined in two ways: (1) 15.00 Å cutoff range, 0.1 charge cutoff, and (2) 30.00 Å cutoff range, 0.05 charge cutoff. Also, the correlation of the binding site openness with the Z component of the electrostatic field for the carbonyl carbon atom was checked.

## Additional Information

**How to cite this article:** Czajlik, A. *et al*. Fine-tuning the extent and dynamics of binding cleft opening as a potential general regulatory mechanism in parvulin-type peptidyl prolyl isomerases. *Sci. Rep.* **7**, 44504; doi: 10.1038/srep44504 (2017).

**Publisher's note:** Springer Nature remains neutral with regard to jurisdictional claims in published maps and institutional affiliations.

## Supplementary Material

Supplementary Information

## Figures and Tables

**Figure 2:**
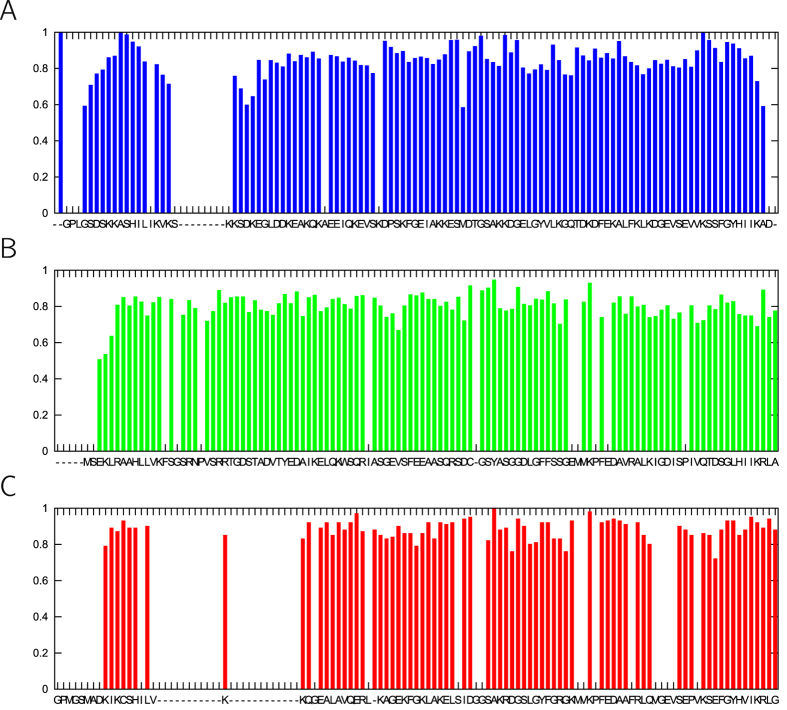
Experimental S^2^ values for the three parvulins investigated. Sequences are aligned to make comparison straightforward (based on the alignment produced by MAMMOTH-Mult, https://ub.cbm.uam.es/software/online/mamothmult.php). (**A**) SaPrsA, (**B**) TbPin1, (**C**) CsPinA.

**Table 1:** Correspondence of the ensembles to experimental data.

	Ensemble size	Backbone RMSD(Å)		Backbone S^2^ correlation	Chemical shift correlation	
		Full molecule	Fit with flexible parts excluded	Full molecule	Cα	Hα
**SaPrsA**		**Residues 1–111**	**Residues 6–111**	**Residues 1–111**		
2JZV	25	2.01 ± 0.65	0.75 ± 0.22	0.59	0.98	0.91
unrestrained	728	2.09 ± 0.57	1.43 ± 0.38	0.58	0.98	0.91
MUMO	728	1.89 ± 0.65	1.12 ± 0.20	0.93	0.99	0.92
**TbPin1**		**Residues 1–115**	**Residues 3–115**	**Residues 1–115**		
2LJ4	20	0.68 ± 0.18	0.43 ± 0.08	0.38	0.96	0.84
unrestrained	728	2.12 ± 0.49	1.93 ± 0.46	0.16	0.97	0.86
MUMO	728	1.71 ± 0.52	1.58 ± 0.53	0.92	0.97	0.86
**CsPinA**		**Residues 1–97**	**Residues 6–97**	**Residues 1–97**		
2RQS	20	2.16 ± 0.72	0.64 ± 0.17	0.11	0.97	0.69
unrestrained	728	3.35 ± 1.00	2.35 ± 0.80	0.23	0.98	0.74
MUMO	728	2.44 ± 0.72	1.21 ± 0.26	0.78*	0.98	0.75

Backbone RMSD values are calculated for all residues with MOLMOL. Note that the number of residues are not comparable and the large values for the deposited ensembles are the consequence of the inclusion of flexible terminal parts. *S2 value for GLY97 excluded.
