# Supplementary material for: Fine-tuning the extent and dynamics of binding cleft opening as a potential general regulatory mechanism in parvulin-type peptidyl prolyl isomerases
Source: Sci Rep. 2017 Mar 16;7:44504. doi: 10.1038/srep44504 (PMC5353683; doi:10.1038/srep44504)
Supplement: Supplementary Information [file srep44504-s1.pdf]

Fine-tuning the extent and dynamics of binding cleft opening as a potential general regulatory mechanism in parvulin-type peptidyl prolyl isomerases

András Czajlik[1], Bertalan Kovács[1], Perttu Permi[2], Zoltán Gáspári[1]

[1]Pázmány Péter Catholic University, Faculty of Information Technology and Bionics, Budapest, Hungary
[2]University of Jyvaskyla, Dept. of Biological and Environmental Science, Dept. of Chemistry, Nanoscience Center, Jyvaskyla, Finland

Supplementary Information

Sources of experimental data
All initial structures and the experimental NOEs were obtained from the Protein Data Bank (PDB) (Supplementary Table S1). Experimental chemical shifts were downloaded from the Biological Magnetic Resonance Data Bank (BMRB). S^2 restraints for SaPrsA were obtained from earlier measurements of one of the coauthors' (P. Permi) goup [1]. Order parameters of CsPinA were taken from the set published in BMRB (ID 11080 [2]), whereas those of TbPin1 were obtained directly from the authors of the original publication where they were reported [3]. Experimental conditions are available in the respective publications [1-3]. Briefly, SaPrsA was measured at 25 °C, in a solution of 20 mM Bis-Tris, pH 6.8 and 8% (v/v) D_2O, with a protein concentration of 1 mM. CsPinA was measured at 16 °C, in a solution of 50 mM Tris, pH 7.5 and 50 mM NaCl, with a protein concentration of 0.8 mM. TbPin1 was measured at 25 °C, in a 20 mM sodium phosphate buffer, pH 7.0, 1 mM DDT, 0.01 mM NaN_3, protein concentration was 0.8-1.0 mM.

Details of the restrained molecular dynamics simulations
Molecular dynamics simulations were performed using the AMBER99SB force field. For each molecule, the first model in the deposited PDB files was used. Hydrogens were removed from the molecules and replaced with appropriate ones in GROMACS (-ignh option in pdb2mx). Histidine protonation states were set as found in the PDB files. For SaPrsA this was equivalent to the protonation states described in Heikkinen et al [1], whereas for TbPin1 and CsPinA protonated side chains were used which were consistent with the available chemical shift and distance restraint lists. Molecules were placed in standard cubic box with minimum distance of 1.5 nm between solute atoms and box wall. After a short initial energy minimization, boxes were filled with water using the TPI3P water model and systems were neutralized with the addition of Cl- ions. Before the production runs, a 1 ns simulation was performed with position restraints applied to the heavy atoms of the proteins.

Supplementary Table S1. Sources of experimental data and parameters of molecular dynamics simulations.

	SaPrsA	TbPin1	CsPinA
Number of Cl- ions	3	1	8
Number of water molecules	10725	9375	9993
Box size (nm^3)	7.02 x 6.70 x 7.3	7.7 x 6.5 x 5.8	7.82 x 4.49 x 6.23
Initial structure and NOEs (PDB ID)	2JZV	2LJ4	2RQS
Chemical shift (BMRB ID)	15628	17918	11080
Order parameters	own data reported in [1]	described in [3], obtained from the authors	from BMRB (11080) [2]

Simulations were performed following the MUMO (Minimal Over-restraining Minimal Under-restraining) protocol described in Richter et al [4]. All 8 replicas were restrained by experimental S^2

values and neighboring replicas were restrained by NOE distances in a pairwise manner. NOE restraining was used as implemented in GROMACS except for averaging of distances between pairwise replicas, whereas S^2 restraining was implemented using the 'half-harmonic' scheme described in Best & Vendruscolo 2004 [5], allowing the system to smoothly approach correspondence to the target experimental parameters using a progress variable. For S^2 calculation at each simulation step a copy of each replica was superimposed to the backbone of the first replica, and after the calculated force vectors were rotated back in accordance of the actual orientation of the given replica [4,6]. These modifications have previously been described [7] and the modified source files for GROMACS 4.5.5 are available at http://users.itk.ppke.hu/~gaszo.

A time step of 1 fs was applied. The cut-off distance for short range neighboring atoms was set to 10 Å. Electrostatic interactions were described by the fast smooth Particle-Mesh Ewald (SPME) electrostatics, with a distance cut-off of 10 Å. Cut-off distance for van der Waals interactions was set to 10 Å as well. All simulations were run at 298 K with temperature coupling ensured by the V-rescale algorithm and a Berendsen barostat (1 bar; NPT ensemble). Distance restraints were applied with a force constant of 30000 $kJ \cdot mol^{-1} \cdot nm^{-2}$, whereas S^2 restraints with 10^8 $kJ \cdot mol^{-1}$.

Analysis of the ligand-binding site

Atoms participating in ligand binding and common to all three investigated structures were identified as follows. Two X-ray structures of Pin1:ligand complexes were used, 1PIN and 3NTP. These two structures contain different ligand types, thus, a set of atoms that can be regarded as common between the two ligands had to be defined first. After visual inspection, 9 such atoms were chosen: from the part corresponding to the proline residue, the ring atoms including the alpha carbon, the amide N, carbonyl C, and from the part corresponding to the preceding residue (Ala in 1PIN), three C atoms corresponding to the C, CA and CB atoms (Supplementary Table S3).

Protein atoms closer than 7 Å to any of these identified 9 ligand atoms were identified and only those common in the two Pin1 structures were retained. In the next step, a MAMMOTH-MULT alignment of the 1PIN, 3NTP, 2JZV, 2LJ4 and 2RQS structures (using the first models from the original NMR ensembles) was generated and based on this mapping, atoms common in all 5 structures were retained. After inspecting the common set of atoms and residues, where the residue type did not match between the structures but the atom type was the same, the atom was retained. Atoms not common between all 5 structures were omitted, the only exception being Cys 113 (1PIN numbering), a residue proposed to be important in catalysis and replaced by Asp in 2JZV and 2RQS, in which cases the Asp CG atom was considered instead of the Cys SG. This resulted in 38 atoms altogether from 10 residues including the two conserved histidines and all residues that were used as a common set of binding site atoms shared by the 5 parvulins. Although this approach contains subjective elements, we expect that the size of the set and the included residues warrant that the results obtained have valid implications.

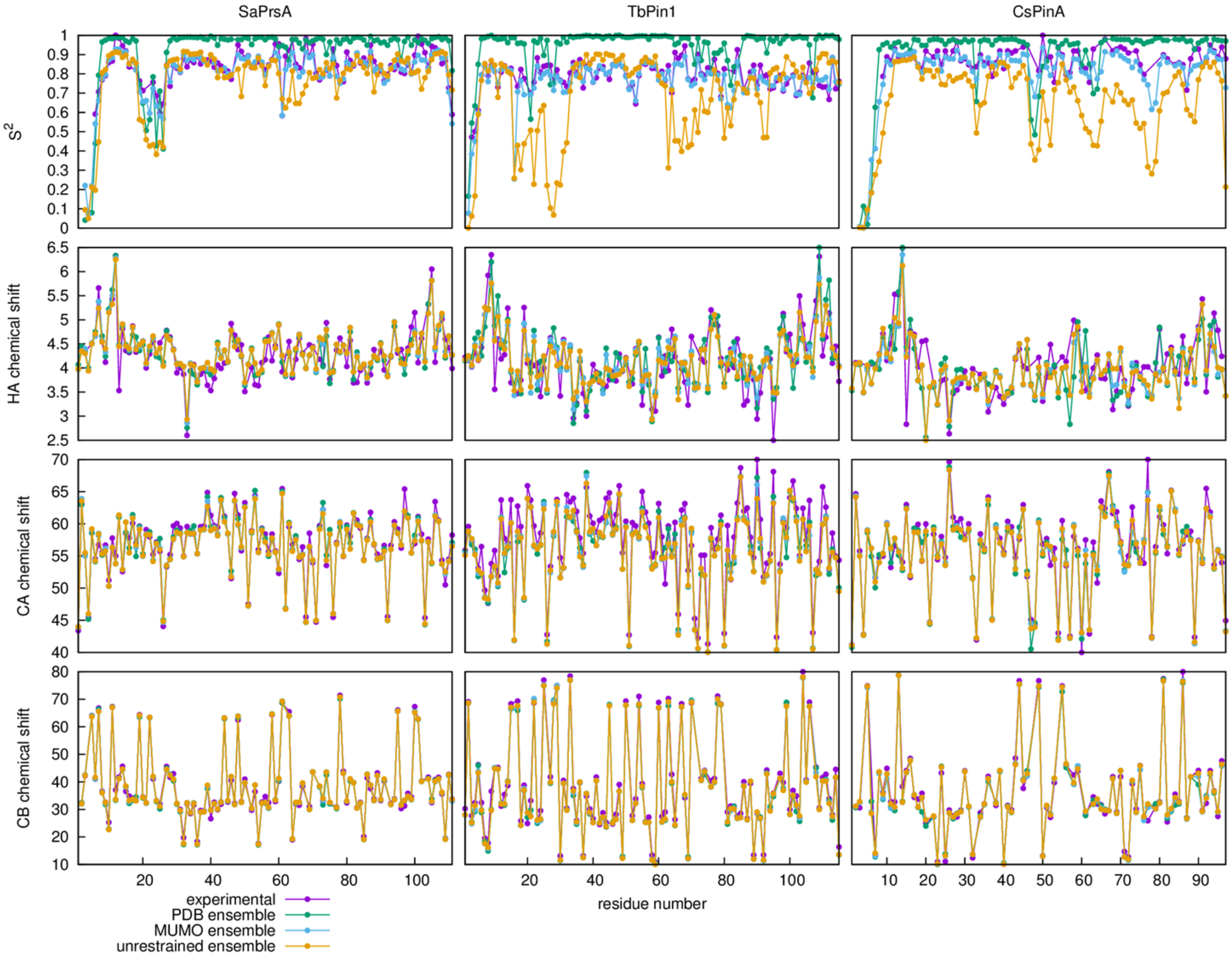

Supplementary Figure S2: Detailed plot of the correlation between experimental and back-calculated order parameters (1st row), and chemical shifts (2-4th row). Chemical shifts were calculated by shiftx2 [8] using default parameters. Correlations of the calculated shifts with experimental ones are reported in Table 1.

Supplementary Table S3. NOE violations in the calculated ensembles.

	SaPrsA	TbPin1	CsPinA
Used for ensemble calculations	1398	1458	1132
Violated above 0.5 A in the restrained ensemble	8	13	13
Violated above 0.5 A in the unrestrained ensemble	71	122	48

Supplementary Table S4. Atom names (1PIN) and atomic numbers of the 9 atoms considered as corresponding to each other in the PDB files 1PIN and 3NTP.

1PIN atom name	Ala CA	Ala CB	Ala C	Pro N	Pro CA	Pro CB	Pro CG	Pro CD	Pro C
1PIN atom number	1216	1219	1217	1220	1221	1224	1225	1226	1222
3NTP atom number	1187	1186	1182	1173	1172	1180	1178	1175	1183

Supplementary Table S5.
Fraction of structural variability described by the PCA modes for selected ensembles. Fractions calculated from the squared weigths reported for each PCA mode in the ProDy nmd files.

PCA mode	MUMO ensembles (Fig4A)	unrestrained ensembles (Fig4B)	binding site residues in the ensembles (Fig4I)	MUMO ensembles with all PPIase domains (Fig5A)
**1**	0.44	0.36	0.42	0.48
**2**	0.25	0.24	0.21	0.23
**3**	0.11	0.12	0.11	0.09
**4**	0.05	0.08	0.06	0.06
**5**	0.04	0.06	0.05	0.03
**6**	0.03	0.04	0.04	0.03
**7**	0.03	0.03	0.03	0.02
**8**	0.02	0.02	0.03	0.02
**9**	0.01	0.02	0.02	0.02
**10**	0.01	0.02	0.02	0.02

Supplementary Figure S6. Sequence alignment of the 100 PPIase domains used in the study. File generated by MAMMOTH-Mult based on the performed structure superposition.

```
2pv2CR1          HILIPLPE----N--------P--------TSDQVNEAESQARAIVDQARN-----G--A
2jzvAR1          HILIKVK-S---K--------K----SDKEG-LDDKEAKQKAEEIQKEVSK-----DP-S
2xp5AR1          ----------------------------------KEEALELINGYIQKIKS-----GE-E
1m5yBR1          --------------------------------DQVNEAESQARAIVDQARN-----G--A
3odkAR1          ---------------------------------TKEEALELINGYIQKIKS-----GE-E
3nrkAR1          --------------------------------------YKEVSEIRKSILA-----DP-S
1jntAR1          --------------------------------HILVKEEKLALDLLEQIKN-------GA
2lj4AR1          HLLVKFS-GSRNPVSRRTG--------DSTADVTYEDAIKELQKWSQRIAS-----GE-V
3jyjAR1          -------------RPSSWRQEQ--------ITRTQEEALELINGYIQKIKS-----GE-E
3ui6AR1          ----------------------------------LCEKHGKIMEAMEKLKS-----G--M
2zr5AR1          -------------RPSSWRQEK--------ITRTKEEALELINGYIQKIKS-----GE-E
3kadAR1          ---------------------------------TKEEALELINGYIQKIKS-----GE-E
1zcnAR1          ----------------------------KIT-RTKEEALELINGYIQKIKS-----GE-E
2zr4AR1          -------------RPSSWRQEK--------ITRTKEEALELINGYIQKIKS-----GE-E
1nmwAR1          HLLVKHS-QSRRPSSWRQ---------EKIT-RTKEEALELINGYIQKIKS-----GE-E
3kahAR1          ---------------------------------TKEEALELINGYIQKIKS-----GE-E
2rqsAR1          --------------------------------HILVKKQGEALAVQERLK------AG-E
1m5yDR2          -----------------------------------EQARVKLEQIAADIKS-----GK-T
2xpbAR1          --------------------------------RTKEEALELINGYIQKIKS-----GE-E
1yw5AR1          HLLIKNN-QSRKPKSWKSP--------DGIS-RTRDESIQILKKHLERILS-----GE-V
3ui5AR1          ---------------------------------ILCEKHGKIMEAMEKLKS-----G--M
2m08AR1          --------------------------------HILVSKQSEALAIMEKLKS-------GE
2mntAR1          HVLVKHK-----DVRRPSSLAPRNKGEKITRSRA--DAINLAQAILAQ-HKERKTWSL-D
2xp8AR1          ---------------------------------TKEEALELINGYIQKIKS-----GE-E
3ikgBR1          -------------RPSSWRQEQ--------ITRTQEEALELINGYIQKIKS-----GE-E
3jyjBR1          -------------RPSSWRQEQ--------ITRTQEEALELINGYIQKIKS-----GE-E
1zk6AR1          ----------------------------------LVADKKTAEEVEKKLKK-----G--E
2kgjAR1          -----------------------------------TKTEDEAKAVLDELNK-------GG
3gpkAR1          --------------------------------ENKPQVFANAEKIVEQLKQ-----G--G
3ikdAR1          -------------RPSSWRQEQ--------ITRTQEEALELINGYIQKIKS-----GE-E
2q5aAR1          ---------------------------------TKEEALELINGYIQKIKS-----GE-E
2pv3AR1          -----PLPE---N--------P--------TSDQVNEAESQARAIVDQARN---------
3rfwAR1          --------------------------------HILVATEKEAKDIINELKGLKGKELD-A
1pinAR1          -----------RPSSWRQ---------EKIT-RTKEEALELINGYIQKIKS-----GE-E
3ik8AR1          -------------RPSSWRQEQ--------ITRTQEEALELINGYIQKIKS-----GE-E
3kceAR1          --------------------------------RTKEEALELINGYIQKIKS-----GE-E
3ikdBR1          ----------------SWRQEQ--------ITRTQEEALELINGYIQKIKS-----GE-E
2xpaAR1          --------------------------------RTKEEALELINGYIQKIKS-----GE-E
3kaiAR1          ----------------------------------KEEALELINGYIQKIKS-----GE-E
2zr6AR1          -------------RPSSWRQEK--------ITRTKEEALELINGYIQKIKS-----GE-E
1m5yDR1          --------------------------------DQVNEAESQARAIVDQARN-----G--A
2pv2DR1          HILIPLPE----N--------P--------TSDQVNEAESQARAIVDQARN-----G--A
2pv2AR1          HILIPLP-E---N--------P--------TSDQVNEAESQARAIVDQARN-----G--A
2itkAR1          ---------------------------------TKEEALELINGYIQKIKS-----GE-E
1f8aBR1          ----------------------SSWRQEKIT-RTKEEALELINGYIQKIKS-----GE-E
3kagAR1          ---------------------------------TKEEALELINGYIQKIKS-----GE-E
2zqvAR1          -------------RPSSWRQEK--------ITRTKEEALELINGYIQKIKS-----GE-E
2xp9AR1          ----------------------------------KEEALELINGYIQKIKS-----GE-E
1eq3AR1          -------------------------------HILCEKHGK-IMEAMEKLKS-------GM
2zqtAR1          -------------RPSSWRQEK--------ITRTKEEALELINGYIQKIKS-----GE-E
3ikgAR1          -------------RPSSWRQEQ--------ITRTQEEALELINGYIQKIKS-----GE-E
1m5yAR2          ----------------------------------DEQARVKLEQIAADIKS-----GK-T
3kafAR1          ---------------------------------TKEEALELINGYIQKIKS-----GE-E
2zquAR1          -------------RPSSWRQEK--------ITRTKEEALELINGYIQKIKS-----GE-E
2zqsAR1          -------------RPSSWRQEK--------ITRTKEEALELINGYIQKIKS-----GE-E
```

```
1j6yAR1          -------------------------GKIILT-TTREAAVEQLKSIREDIVS-----GK-A
2xp6AR1          ----------------------------------KEEALELINGYIQKIKS-----GE-E
2rudAR1          HLLVKHSQS---RRPSSWRQEK--------ITRTKEEALELINGYIQKIKS-----GE-E
3kacBR1          SR-----------RPSSWRQEQ--------ITRTQEEALELINGYIQKIKS-----GE-E
3ik8BR1          ----------------SWRQEQ--------ITRTQEEALELINGYIQKIKS-----GE-E
3ntpAR1          -------------------------------TRTKEEALELINGYIQKIKS-----GE-E
2m1iAR1          --------------------------------HILVKKQGEALAVQERLKA-------GE
2pv1AR1          HILIPLP-E---N--------P--------TSDQVNEAESQARAIVDQARN-----G--A
1nmvAR1          HLLVKHS-QSRRPSSWRQ---------EKIT-RTKEEALELINGYIQKIKS-----GE-E
1m5yAR1          -------------------------------SDQVNEAESQARAIVDQARN-----G--A
3i6cAR1          -------------RPSSWRQEQ--------ITRTQEEALELINGYIQKIKS-----GE-E
1fjdAR1          --------------------------------HILCEKHGKIMEAMEKLKS-------GM
1m5yCR2          --------------------------------------------IAADIKS-----GK-T
3i6cBR1          -------------RPSSWRQEQ--------ITRTQEEALELINGYIQKIKS-----GE-E
3wh0AR1          ---------------------K--------ITRTKEEALELINGYIQKIKS-----GE-E
3ui4AR1          -------------------------------------KHGKIMEAMEKLKS-----G--M
3kacAR1          -------------RPSSWRQEQ--------ITRTQEEALELINGYIQKIKS-----GE-E
2xp7AR1          ---------------------------------TKEEALELINGYIQKIKS-----GE-E
3gpkBR1          ---------------------A--------TEENKPQVFANAEKIVEQLKQ-----G--G
3tdbAR1          ---------------SSWRQEK--------ITRTKEEALELINGYIQKIKS-----GE-E
2rucAR1          HLLVKHSQS---RRPSSWRQEK--------ITRTKEEALELINGYIQKIKS-----GE-E
1jnsAR1          --------------------------------HILVKEEKLALDLLEQIKN-------GA
2f21AR1          ----------RRPSSWRQ---------EKIT-RTKEEALELINGYIQKIKS-----GE-E
2pv3BR1          -----PLPE---N--------P--------TSDQVNEAESQARAIVDQARN---------
1m5yCR1          --------------------------------DQVNEAESQARAIVDQARN-----G--A
3oobAR1          -----------------------------------EEALELINGYIQKIKS-----GE-E
3tc5AR1          ---------------------------------TKEEALELINGYIQKIKS-----GE-E
2pv2BR1          HILIPLP-E---N--------P--------TSDQVNEAESQARAIVDQARN-----G--A
2xp3AR1          ---------------------------------TKEEALELINGYIQKIKS-----GE-E
3tczAR1          ---------------SSWRQEK--------ITRTKEEALELINGYIQKIKS-----GE-E
3kabAR1          ---------------------------------TKEEALELINGYIQKIKS-----GE-E
1m5yBR2          -------------------------------------------QIAADIKS-----GK-T
2xp4AR1          -----------------------------------EEALELINGYIQKIKS-----GE-E
4u86AR1          -----------------------------------------------KIKS-----GE-E
4tnsBR1          --------------------------------------------------S-----GE-E
4g2pAR1          -------LLK-----------P--------SPINDQQARLKLEEIAADIKS-----GK-T
4tyoAR1          --RPSSWRQE-----------Q--------ITRTQEEALELINGYIQKIKS-----GE-E
4tnsAR1          --------------------------------------------------------GE-E
4u85AR1          -----------------------------------------------KIKS-----GE-E
4g2pBR1          -------LLK-----------P--------SPINDQQARLKLEEIAADIKS-----GK-T
4tyoBR1          SRRPSSWRQE-----------Q--------ITRTQEEALELINGYIQKIKS-----GE-E
4qibAR1          ------WRQE-----------K--------ITRTKEEALELINGYIQKIKS-----GE-E
4wo7AR1          -------------------------------------DKKTAEEVEKKLKK-----G--E
4wo7BR1          --------------------------------------KKTAEEVEKKLKK-----G--E
4u84AR1          -----------------------------------------------KIKS-----GE-E


2pv2CR1          DFGKLAIAHSAD-Q-QALNGGQMGWGR---IQ-ELPGIFAQALSTAKKG-----DIV-G-
2jzvAR1          KFGEIAKKESMDTG-SAKKDGELGYVL---KG-QTDKDFEKALFKLKDG-----EVS-E-
2xp5AR1          DFESLASQFSDC-S-SAKARGDLGAFS---RG-QMQKPFEDASFALRTG-----EMS-G-
1m5yBR1          DFGKLAIAHSADQ--QALNGGQMGWGR---IQ-ELPGIFAQALSTAKKG-----DIV-G-
3odkAR1          DFESLASQFSDC-S-SAKARGDLGAFS---RG-QMQKPFEDASFALRTG-----EMS-G-
3nrkAR1          SFALIAGSPRNDPA-LRARRGMVEWISSFDLY-KYSKITATIAAPLPNG-----GVS--E
1jntAR1          DFGKLAKKHSICPS--GKRGGDLGEFR---QG-QMVPAFDKVVFSCPVL-----EPT-G-
2lj4AR1          SFEEAASQRSDC-G-SYASGGDLGFFS---SG-EMMKPFEDAVRALKIG-----DIS-P-
3jyjAR1          DFESLASQFSDC-S-SAKARGDLGAFS---RG-QMQKPFEDASFALRTG-----EMS-G-
3ui6AR1          RFNEVAAQYSED---KARQGGDLGWMT---RG-SMVGPFQEAAFALPVSGMDKPVFT-DP
2zr5AR1          DFESLASQFSDC-S-SAKARGDLGAFS---RG-QMQKPFEDASFALRTG-----EMS-G-
3kadAR1          DFESLASQFSDC-S-SAKARGDLGAFS---RG-QMAKPFEDASFALRTG-----EMS-G-
```

```
1zcnAR1        DFESLASQFSDC-S-SAKARGDLGAFS---RG-QMQKPFEDASFALRTG-----EMS-G-
2zr4AR1        DFESLASQFSDC-S-SAKARGDLGAFS---RG-QMQKPFEDASFALRTG-----EMS-G-
1nmwAR1        DFESLASQFSDC-S-SAKARGDLGAFS---RG-QMQKPFEDASFALRTG-----EMS-G-
3kahAR1        DFESLASQFSDC-S-SAKARGDLGAFS---RG-QMQKPFEDASFALRTG-----EMS-G-
2rqsAR1        KFGKLAKELSIDGG-SAKRDGSLGYFG---RG-KMVKPFEDAAFRLQVG-----EVS-E-
1m5yDR2        TFAAAAKEFSQDPG-SANQGGDLGWAT---PD-IFDPAFRDALTRLNKG-----QMS-A-
2xpbAR1        DFESLASQFSDC-S-SAKARGDLGAFS---RG-QMQKPFEDASFALRTG-----EMS-G-
1yw5AR1        KLSELANTESDC-S-SHDRGGDLGFFS---KG-QMQPPFEEAAFNLHVG-----EVS-N-
3ui5AR1        RFNEVAAQYSED---KARQGGDLGWMT---RG-SMVGPFQEAAFALPVSGMDKPVFT-DP
2m08AR1        KFGKLAKELSIDSG-SAKKNGNLGYFT---KG-MMVKPFEDAAFKLQVG-----EVS-E-
2mntAR1        EFVQVVRDFSEC-G-SAKRDGDLGMVE---SG-TYTEGFDTVAFSLKSG-----EVS-A-
2xp8AR1        DFESLASQFSDC-S-SAKARGDLGAFS---RG-QMQKPFEDASFALRTG-----EMS-G-
3ikgBR1        DFESLASQFSDC-S-SAKARGDLGAFS---RG-QMQKPFEDASFALRTG-----EMS-G-
3jyjBR1        DFESLASQFSDC-S-SAKARGDLGAFS---RG-QMQKPFEDASFALRTG-----EMS-G-
1zk6AR1        KFEDLAKEYSTDS--SASKGGDLGWFA---KEGQMDETFSKAAFKLKTG-----EVS-D-
2kgjAR1        DFAALAKEKSADII-SARNGGDMGWLE---DA-TIPDELKNAG-LKEKG-----QLS-G-
3gpkAR1        SFVAYARQYSEA-S-TAAVGGDLGWIR---LA-QLPTELATTAAS-GPG-----QLA-G-
3ikdAR1        DFESLASQFSDC-S-SAKARGDLGAFS---RG-QMQKPFEDASFALRTG-----EMS-G-
2q5aAR1        DFESLASQFSDC-S-SAKARGDLGAFS---RG-QMQKPFEDASFALRTG-----EMS-G-
2pv3AR1        DFGKLAIAHSAD-Q-QALNGGQMGWGR---IQ-ELPGIFAQALSTAKKG-----DIV-G-
3rfwAR1        KFSELAKEKSIDPG-SKNQGGELGWFD---QS-TMVKPFTDAAFALKNG-----TIT-TT
1pinAR1        DFESLASQFSDC-S-SAKARGDLGAFS---RG-QMQKPFEDASFALRTG-----EMS-G-
3ik8AR1        DFESLASQFSDC-S-SAKARGDLGAFS---RG-QMQKPFEDASFALRTG-----EMS-G-
3kceAR1        DFESLASQFSDC-S-SAKARGDLGAFS---RG-QMQKPFEDASFALRTG-----EMS-G-
3ikdBR1        DFESLASQFSDC-S-SAKARGDLGAFS---RG-QMQKPFEDASFALRTG-----EMS-G-
2xpaAR1        DFESLASQFSDC-S-SAKARGDLGAFS---RG-QMQKPFEDASFALRTG-----EMS-G-
3kaiAR1        DFESLASQFSDC-S-SAKARGDLGAFS---RG-QMQKPFEDASFALRTG-----EMS-G-
2zr6AR1        DFESLASQFSDC-S-SAKARGDLGAFS---RG-QMQKPFEDASFALRTG-----EMS-G-
1m5yDR1        DFGKLAIAHSADQ--QALNGGQMGWGR---IQ-ELPGIFAQALSTAKKG-----DIV-G-
2pv2DR1        DFGKLAIAHSAD-Q-QALNGGQMGWGR---IQ-ELPGIFAQALSTAKKG-----DIV-G-
2pv2AR1        DFGKLAIAHSAD-Q-QALNGGQMGWGR---IQ-ELPGIFAQALSTAKKG-----DIV-G-
2itkAR1        DFESLASQFSDC-S-SAKARGDLGAFS---RG-QMQKPFEDASFALRTG-----EMS-G-
1f8aBR1        DFESLASQFSDC-S-SAKARGDLGAFS---RG-QMQKPFEDASFALRTG-----EMS-G-
3kagAR1        DFESLASQFSDC-S-SAKARGDLGAFS---RG-QMQKPFEDASFALRTG-----EMS-G-
2zqvAR1        DFESLASQFSDC-S-SAKARGDLGAFS---RG-QMQKPFEDASFALRTG-----EMS-G-
2xp9AR1        DFESLASQFSDC-S-SAKARGDLGAFS---RG-QMQKPFEDASFALRTG-----EMS-G-
1eq3AR1        RFNEVAAQYSED---KARQGGDLGWMT---RG-SMVGPFQEAAFALPVSGMDKPVFTDP-
2zqtAR1        DFESLASQFSDC-S-SAKARGDLGAFS---RG-QAQKPFEDASFALRTG-----EMS-G-
3ikgAR1        DFESLASQFSDC-S-SAKARGDLGAFS---RG-QMQKPFEDASFALRTG-----EMS-G-
1m5yAR2        TFAAAAKEFSQDPG-SANQGGDLGWAT---PD-IFDPAFRDALTRLNKG-----QMS-A-
3kafAR1        DFESLASQFSDC-S-SAKARGDLGAFS---RG-QMAKPFEDASFALRTG-----EMS-G-
2zquAR1        DFESLASQFSDC-S-SAKARGDLGAFS---RG-QMQKPFEDASFALRTG-----EMS-G-
2zqsAR1        DFESLASQFSDA-S-SAKARGDLGAFS---RG-QMQKPFEDASFALRTG-----EMS-G-
1j6yAR1        NFEEVATRVSDC-S-SAKRGGDLGSFG---RG-QMQKPFEEATYALKVG-----DIS-D-
2xp6AR1        DFESLASQFSDC-S-SAKARGDLGAFS---RG-QMAKPFEDASFALRTG-----EMS-G-
2rudAR1        DFESLASQFSDD-S-SAKARGDLGAFS---RG-QMQKPFEDASFALRTG-----EMS-G-
3kacBR1        DFESLASQFSDC-S-SAKARGDLGAFS---RG-QMQKPFEDASFALRTG-----EMS-G-
3ik8BR1        DFESLASQFSDC-S-SAKARGDLGAFS---RG-QMQKPFEDASFALRTG-----EMS-G-
3ntpAR1        DFESLASQFSDC-S-SAKARGDLGAFS---RG-QMQKPFEDASFALRTG-----EMS-G-
2m1iAR1        KFGKLAKELSIDGG-SAKRDGSLGYFG---RG-KMVKPFEDAAFRLQVG-----EVS-E-
2pv1AR1        DFGKLAIAHSAD-Q-QALNGGQMGWGR---IQ-ELPGIFAQALSTAKKG-----DIV-G-
1nmvAR1        DFESLASQFSDC-S-SAKARGDLGAFS---RG-QMQKPFEDASFALRTG-----EMS-G-
1m5yAR1        DFGKLAIAHSADQ--QALNGGQMGWGR---IQ-ELPGIFAQALSTAKKG-----DIV-G-
3i6cAR1        DFESLASQFSDC-S-SAKARGDLGAFS---RG-QMQKPFEDASFALRTG-----EMS-G-
1fjdAR1        RFNEVAAQYSED---KARQGGDLGWMT---RG-SMVGPFQEAAFALPVSGMDKPVFTDP-
1m5yCR2        TFAAAAKEFSQDPG-SANQGGDLGWAT---PD-IFDPAFRDALTRLNKG-----QMS-A-
3i6cBR1        DFESLASQFSDC-S-SAKARGDLGAFS---RG-QMQKPFEDASFALRTG-----EMS-G-
3wh0AR1        DFESLASQFSDC-S-SAKARGDLGAFS---RG-QMQKPFEDASFALRTG-----EMS-G-
3ui4AR1        RFNEVAAQYSED---KARQGGDLGWMT---RG-SMVGPFQEAAFALPVSGMDKPVFT-DP
```

```
3kacAR1         DFESLASQFSDC-S-SAKARGDLGAFS---RG-QMQKPFEDASFALRTG-----EMS-G-
2xp7AR1         DFESLASQFSDC-S-SAKARGDLGAFS---RG-QMQKPFEDASFALRTG-----EMS-G-
3gpkBR1         SFVAYARQYSEA-S-TAAVGGDLGWIR---LA-QLPTELATTAAS-GPG-----QLA-G-
3tdbAR1         DFESLASQFSDC-S-SAKARGDLGAFS---RG-QMQKPFEDASFALRTG-----EMS-G-
2rucAR1         DFESLASQFSDC-S-SAKARGDLGAFS---RG-QMQKPFEDASFALRTG-----EMS-G-
1jnsAR1         DFGKLAKKHSICPS--GKRGGDLGEFR---QG-QMVPAFDKVVFSCPVL-----EPT-G-
2f21AR1         DFESLASQFSDC-S-SAKARGDLGAFS---RG-QMQKPFEDASFALRTG-----EMS-G-
2pv3BR1         DFGKLAIAHSAD-Q-QALNGGQMGWGR---IQ-ELPGIFAQALSTAKKG-----DIV-G-
1m5yCR1         DFGKLAIAHSADQ--QALNGGQMGWGR---IQ-ELPGIFAQALSTAKKG-----DIV-G-
3oobAR1         DFESLASQFSDC-S-SAKARGDLGAFS---RG-QMQKPFEDASFALRTG-----EMS-G-
3tc5AR1         DFESLASQFSDC-S-SAKARGDLGAFS---RG-QMQKPFEDASFALRTG-----EMS-G-
2pv2BR1         DFGKLAIAHSAD-Q-QALNGGQMGWGR---IQ-ELPGIFAQALSTAKKG-----DIV-G-
2xp3AR1         DFESLASQFSDC-S-SAKARGDLGAFS---RG-QMQKPFEDASFALRTG-----EMS-G-
3tczAR1         DFESLASQFSDC-S-SAKARGDLGAFS---RG-QMQKPFEDASFALRTG-----EMS-G-
3kabAR1         DFESLASQFSDC-S-SAKARGDLGAFS---RG-QMQKPFEDASFALRTG-----EMS-G-
1m5yBR2         TFAAAAKEFSQDPG-SANQGGDLGWAT---PD-IFDPAFRDALTRLNKG-----QMS-A-
2xp4AR1         DFESLASQFSDC-S-SAKARGDLGAFS---RG-QMQKPFEDASFALRTG-----EMS-G-
4u86AR1         DFESLASQFSD---SSAKARGDLGAFS---RG-QMQKPFEDASFALRTG-----EMS-G-
4tnsBR1         DFESLASQFSDC-S-SAKARGDLGAFS---RG-QMQKPFEDASFALRTG-----EMS-G-
4g2pAR1         TFAAAAKEYSQD-PGSANQGGDLGWAT---PD-IFDPAFRDALTKLHKG-----QIS-A-
4tyoAR1         DFESLASQFSDC-S-SAKARGDLGAFS---RG-QMQKPFEDASFALRTG-----EMS-G-
4tnsAR1         DFESLASQFSDC-S-SAKARGDLGAFS---RG-QMQKPFEDASFALRTG-----EMS-G-
4u85AR1         DFESLASQFSD---SSAKARGDLGAFS---RG-QMQKPFEDASFALRTG-----EMS-G-
4g2pBR1         TFAAAAKEYSQD-PGSANQGGDLGWAT---PD-IFDPAFRDALTKLHKG-----QIS-A-
4tyoBR1         DFESLASQFSDC-S-SAKARGDLGAFS---RG-QMQKPFEDASFALRTG-----EMS-G-
4qibAR1         DFESLASQFSD--S-SAKARGDLGAFS---RG-QMQKPFEDASFALRTG-----EMS-G-
4wo7AR1         KFEDLAKEYSTD-S-SASKGGDLGWFA---KE-GQDETFSKAAFKLKTG-----EVS-D-
4wo7BR1         KFEDLAKEYSTD-S-SASKGGDLGWFA---KE-GQDETFSKAAFKLKTG-----EVS-D-
4u84AR1         DFESLASQFSD---SSAKARGDLGAFS---RG-QMQKPFEDASFALRTG-----EMS-G-


2pv2CR1         PIRS-GVGFHILKVND------------------------
2jzvAR1         VVKS-SFGYHIIKAD-------------------------
2xp5AR1         PVFT-DSGIHIILRTE------------------------
1m5yBR1         PIRS-GVGFHILKVNDLRTEVHARHILL------------
3odkAR1         PVFT-DSGIHIILRTE------------------------
3nrkAR1         VFRDERKRYCILKIEGKRPTPMENLR--------------
1jntAR1         PLHT-QFGYHIIKV--------------------------
2lj4AR1         IVQT-DSGLHIIKR--------------------------
3jyjAR1         PVFT-DSGIHIILRTE------------------------
3ui6AR1         PVKT-KFGYHIIMVEGRK----------------------
2zr5AR1         PVFT-DSGIHIILRTE------------------------
3kadAR1         PVFT-DSGIHIILRTE------------------------
1zcnAR1         PVFT-DSGIHIILRTE------------------------
2zr4AR1         PVFT-DSGIHIILRTE------------------------
1nmwAR1         PVFT-DSGIHIILRT-------------------------
3kahAR1         PVFT-DSGIHIILRTE------------------------
2rqsAR1         PVKS-EFGYHVIKR--------------------------
1m5yDR2         PVHS-SFGWHLIELL-DTRNVDKRAYRM------------
2xpbAR1         PVFT-DSGIHIILRTE------------------------
1yw5AR1         IIET-NSGVHILQRT-------------------------
3ui5AR1         PVKT-KFGYHIIMVEGRK----------------------
2m08AR1         PIKS-EFGYHIIKR--------------------------
2mntAR1         PVET-ELGVHLIYRV-------------------------
2xp8AR1         PVFT-DSGIHIILRTE------------------------
3ikgBR1         PVFT-DSGIHIILRTE------------------------
3jyjBR1         PVFT-DSGIHIILRTE------------------------
1zk6AR1         PVKT-QYGYHIIKKTEE-----------------------
2kgjAR1         VIKS-SVGFLIVRLDD------------------------
```

```
3gpkAR1          PVEI-RGGFSILYLIDKREGH-------------------
3ikdAR1          PVFT-DSGIHIILRTE------------------------
2q5aAR1          PVFT-DSGIHIILRTE------------------------
2pv3AR1          PIRS-GVGFHILKVNDLAAQKD------------------
3rfwAR1          PVKT-NFGYHVIL---------------------------
1pinAR1          PVFT-DSGIHIILRTE------------------------
3ik8AR1          PVFT-DSGIHIILRTE------------------------
3kceAR1          PVFT-DSGIHIILRTE------------------------
3ikdBR1          PVFT-DSGIHIILRTE------------------------
2xpaAR1          PVFT-DSGIHIILRTE------------------------
3kaiAR1          PVFT-DSGIHIILRTE------------------------
2zr6AR1          PVFT-DSGIHIILRTE------------------------
1m5yDR1          PIRS-GVGFHILKVNDLRGESKNISVTE------------
2pv2DR1          PIRS-GVGFHILKVND------------------------
2pv2AR1          PIRS-GVGFHILKVND------------------------
2itkAR1          PVFT-DSGIHIILRTE------------------------
1f8aBR1          PVFT-DSGIHIILRTE------------------------
3kagAR1          PVFT-DSGIHIILRTE------------------------
2zqvAR1          PVFT-DSGIHIILRTE------------------------
2xp9AR1          PVFT-DSGIHIILRTE------------------------
1eq3AR1          PVKT-KFGYHIIMVE-------------------------
2zqtAR1          PVFT-DSGIHIILRTE------------------------
3ikgAR1          PVFT-DSGIHIILRTE------------------------
1m5yAR2          PVHS-SFGWHLIELL-DTRNVD-RAYRM------------
3kafAR1          PVFT-DSGIHIILRTE------------------------
2zquAR1          PVFT-DSGIHIILRTE------------------------
2zqsAR1          PVFT-DSGIHIILRTE------------------------
1j6yAR1          IVDT-DSGVHIIKRTA------------------------
2xp6AR1          PVFT-DSGIHIILRTE------------------------
2rudAR1          PVFT-DSGIHIILRT-------------------------
3kacBR1          PVFT-DSGIHIILRTE------------------------
3ik8BR1          PVFT-DSGIHIILRTE------------------------
3ntpAR1          PVFT-DSGIHIILRTE------------------------
2m1iAR1          PVKS-EFGYHVIKR--------------------------
2pv1AR1          PIRS-GVGFHILKVND------------------------
1nmvAR1          PVFT-DSGIHIILRT-------------------------
1m5yAR1          PIRS-GVGFHILKVNDLRGESKNISVT-------------
3i6cAR1          PVFT-DSGIHIILRTE------------------------
1fjdAR1          PVKT-KFGYHIIMVE-------------------------
1m5yCR2          PVHS-SFGWHLIELL-DTRN---RAYRMLMNRKFSEEAAS
3i6cBR1          PVFT-DSGIHIILRTE------------------------
3wh0AR1          PVFT-DSGIHIILRTE------------------------
3ui4AR1          PVKT-KFGYHIIMVEGRK----------------------
3kacAR1          PVFT-DSGIHIILRTE------------------------
2xp7AR1          PVFT-DSGIHIILRTE------------------------
3gpkBR1          PVEI-RGGFSILYLIDKR----------------------
3tdbAR1          PVFT-DSGIHIILRTE------------------------
2rucAR1          PVFT-DSGIHIILRT-------------------------
1jnsAR1          PLHT-QFGYHIIKV--------------------------
2f21AR1          PVFT-DSGIHIILRTE------------------------
2pv3BR1          PIRS-GVGFHILKVNDLAAQKD------------------
1m5yCR1          PIRS-GVGFHILKVNDL-TEVHARHILLK-----------
3oobAR1          PVFT-DSGIHIILRTE------------------------
3tc5AR1          PVFT-DSGIHIILRTE------------------------
2pv2BR1          PIRS-GVGFHILKVND------------------------
2xp3AR1          PVFT-DSGIHIILRTE------------------------
3tczAR1          PVFT-DSGIHIILRTE------------------------
3kabAR1          PVFT-DSGIHIILRTE------------------------
1m5yBR2          PVHS-SFGWHLIELL-DTRN---RAYRMLMNRKFSEEAA-
```

```
2xp4AR1          PVFT-DSGIHIILRTE------------------------
4u86AR1          PVFT-DSGIHIILRTE------------------------
4tnsBR1          PVFT-DSGIHIILRTE------------------------
4g2pAR1          PVHS-SFGWHLIELLDTRKV--------------------
4tyoAR1          PVFT-DSGIHIILRTE------------------------
4tnsAR1          PVFT-DSGIHIILRTE------------------------
4u85AR1          PVFT-DSGIHIILRTE------------------------
4g2pBR1          PVHS-SFGWHLIELLDTRK---------------------
4tyoBR1          PVFT-DSGIHIILRTE------------------------
4qibAR1          PVFT-DSGIHIILRTE------------------------
4wo7AR1          PVKT-QYGYHIIKKTEERGKY-------------------
4wo7BR1          PVKT-QYGYHIIKKTEERGKYD------------------
4u84AR1          PVFT-DSGIHIILRTE------------------------
```

Supplementary references

1. Heikkinen, O. *et al.* Solution structure of the parvulin-type PPIase domain of Staphylococcus aureus PrsA--implications for the catalytic mechanism of parvulins. *BMC Struct. Biol.* **9,** 17 (2009).

2. Jaremko, Ł. *et al.* Structure and dynamics of the first archaeal parvulin reveal a new functionally important loop in parvulin-type prolyl isomerases. *J. Biol. Chem.* **286,** 6554–6565 (2011).

3. Sun, L. *et al.* Solution structural analysis of the single-domain parvulin TbPin1. *PLoS One* **7,** e43017 (2012).

4. Richter, B., Gsponer, J., Várnai, P., Salvatella, X. & Vendruscolo, M. The MUMO (minimal under-restraining minimal over-restraining) method for the determination of native state ensembles of proteins. *J. Biomol. NMR* **37,** 117–135 (2007).

5. Best, R. B. & Vendruscolo, M. Determination of protein structures consistent with NMR order parameters. *J. Am. Chem. Soc.* **126,** 8090–8091 (2004).

6. Gáspári, Z., Várnai, P., Szappanos, B. & Perczel, A. Reconciling the lock-and-key and dynamic views of canonical serine protease inhibitor action. *FEBS Lett.* **584,** 203–206 (2010).

7. Fizil, Á., Gáspári, Z., Barna, T., Marx, F. & Batta, G. 'Invisible' conformers of an antifungal disulfide protein revealed by constrained cold and heat unfolding, CEST-NMR experiments, and molecular dynamics calculations. *Chem. - A Eur. J.* **21,** 5136–5144 (2015).

8. Han, B., Liu, Y., Ginzinger, S. W. & Wishart, D. S. SHIFTX2: Significantly improved protein chemical shift prediction. *J. Biomol. NMR* **50,** 43–57 (2011).
